# Molecular Hybridization of Alkaloids Using 1,2,3-Triazole-Based Click Chemistry

**DOI:** 10.3390/molecules28227593

**Published:** 2023-11-14

**Authors:** Devan Buchanan, Ashley M. Pham, Sandeep K. Singh, Siva S. Panda

**Affiliations:** 1 Department of Chemistry and Biochemistry, Augusta University, Augusta, GA 30912, USA; devanbuchanan@gmail.com (D.B.); aspham@augusta.edu (A.M.P.); 2 Jindal Global Business School, OP Jindal Global University, Sonipat 131001, India; sandeepk.singh@jgu.edu.in; 3 Department Biochemistry and Molecular Biology, Augusta University Augusta, GA 30912, USA

**Keywords:** heterocycles, 1,2,3-triazole, alkaloids, biological properties, molecular hybridization, click chemistry, ADMET properties

## Abstract

Alkaloids found in multiple species, known as ‘driver species’, are more likely to be included in early-stage drug development due to their high biodiversity compared to rare alkaloids. Many synthetic approaches have been employed to hybridize the natural alkaloids in drug development. Click chemistry is a highly efficient and versatile reaction targeting specific areas, making it a valuable tool for creating complex natural products and diverse molecular structures. It has been used to create hybrid alkaloids that address their limitations and serve as potential drugs that mimic natural products. In this review, we highlight the recent advancements made in modifying alkaloids using click chemistry and their potential medicinal applications. We discuss the significance, current trends, and prospects of click chemistry in natural product-based medicine. Furthermore, we have employed computational methods to evaluate the ADMET properties and drug-like qualities of hybrid molecules.

## 1. Introduction

### 1.1. Natural Products as a Source of Bioactive Compounds

Natural products (NPs) are a valuable source of therapeutic substances due to their intricate and varied structures [1,2,3]. NPs have been instrumental in drug discovery, especially in treating cancer and infectious diseases [4,5,6]. Their diverse stereo centers, sp3 carbon, and labile functionalities have been instrumental. NPs have also been effective in treating other medical conditions such as cardiovascular diseases (such as statins) and multiple sclerosis (such as fingolimod) [7,8,9]. Their larger molecular mass, higher number of sp3 carbon and oxygen atoms, and lower number of nitrogen and halogen atoms contribute to their unique molecular scaffolds, offering a wealth of structural diversity for drug development [10,11]. Over 60% of the drugs on the market are derived from NPs, highlighting their importance in the pharmaceutical industry [12]. However, the complex nature of NPs often requires complicated synthetic strategies and time-consuming multistep syntheses, resulting in limited yields and derivatives. Therefore, simple, modular, selective, and reliable chemistry is necessary for the late-stage functionalization and diversification of natural products. Alkaloids are an essential class of compounds found in natural products.

Alkaloids are small organic molecules that contain nitrogen, usually in a ring, and are present in plants (about 20% of plant species) [13,14,15]. They have a noticeable impact on the physiology of both humans and animals. Common examples of alkaloids used as drugs include atropine, berberine, cocaine, codeine, capsaicin, ephedrine, morphine, nicotine, quinine, papaverine, reserpine, strychnine, vinblastine, vincristine, etc., and are known for their diverse biological properties [16,17,18,19]. Modifying the chemical structure of known alkaloids is an essential component of drug discovery, as it enables researchers to study the relationship between structure and activity and uncover new biological responses [20].

**Figure 1:**
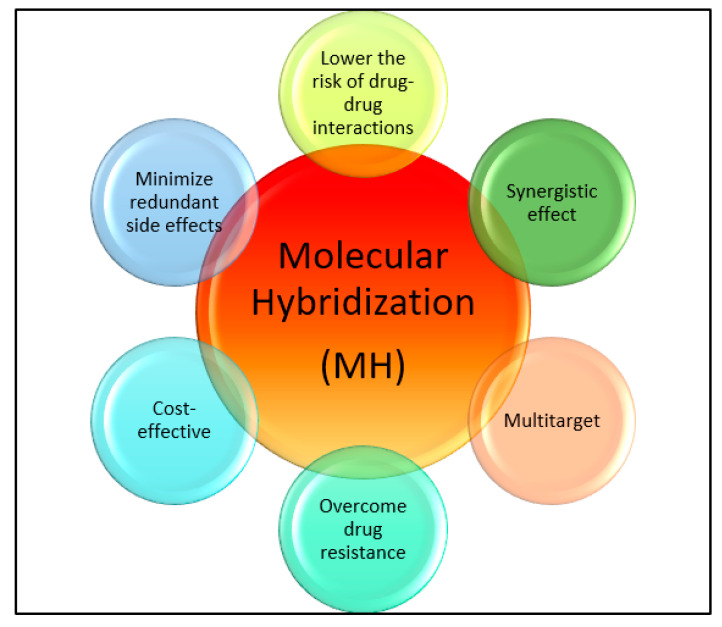
Importance of molecular hybridization.

Drug discovery in modern times involves molecular hybridization, which is used for rationally designing drugs [21,22,23]. This method combines different pharmacophoric moieties of bioactive substances covalently, creating new hybrid compounds that can be more effective than their parent pharmacophores. Hybridizing various organic molecular motifs with different pharmacological activities can create compounds with modified selectivity profiles, different modes of action, reduced side effects, the ability to overcome multidrug resistance, and an improved safety profile [24,25,26,27,28,29,30,31]. This approach helps with the design of multifunctional drug candidates and can overcome the limitations associated with current drugs by providing various advantages (see Figure 1).

### 1.2. Click Chemistry: Principles and Applications

In 2001, Sharpless and his team introduced the concept of click chemistry, which is now widely used in the scientific community. Click chemistry is a simple and efficient way to join two molecular building blocks under mild and adaptable conditions, resulting in high yields while requiring minimal purification. This method is user-friendly and can be compared to fastening two seatbelt ends together. The versatility of click chemistry has made it an indispensable tool in various fields such as analytical chemistry, materials science, chemical biology, and drug development [32,33,34].

Click chemistry involves merging small molecular units to form new molecular attributes. Nevertheless, individuals in the scientific and engineering fields, who need to possess the proper skills and equipment to execute such connecting operations with consistency, can face difficulties in this domain. Fortunately, the chemical strategies available today, among them click chemistry, simplify the process of molecular connectivity with innovative methods continuously emerging [35].

**Figure 2:**
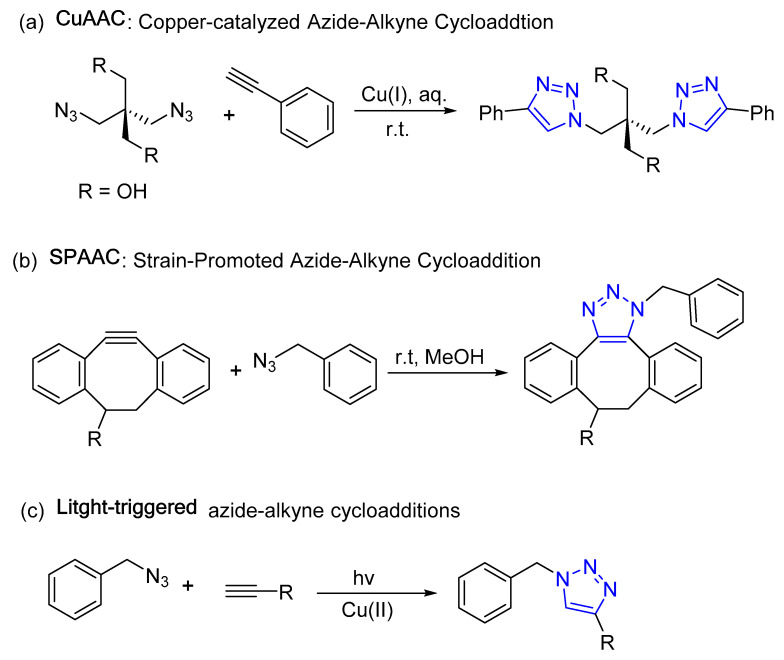
Representative 1,2,3-triazole-based click chemistry reactions.

A “click reaction” is simply a reaction forming new carbon–heteroatom bonds in high yields and selectivity. The term “click” was later coined to indicate the ease of execution, and is synonymous with buckling (clicking) a seatbelt. The most widely used click reaction in medicinal chemistry and biochemistry is the formation of a 1,2,3-triazole moiety by combination of an azide and alkyne. Specific examples that form the 1,2,3-triazole functionality include copper-catalyzed azide–alkyne cycloaddition (CuAAC), strain-promoted azide–alkyne cycloaddition (SPAAC), photoinduced click reactions (light-triggered), and bioorthogonal click chemistry reactions [36,37,38,39,40,41,42]. These reactions are defined as 1,3-dipolar cycloadditions between an alkyne and azide functional group, resulting in a 1,4-disubstituted 1,2,3-triazole as a linker between the two coupled units (Figure 2).

**Figure 3:**
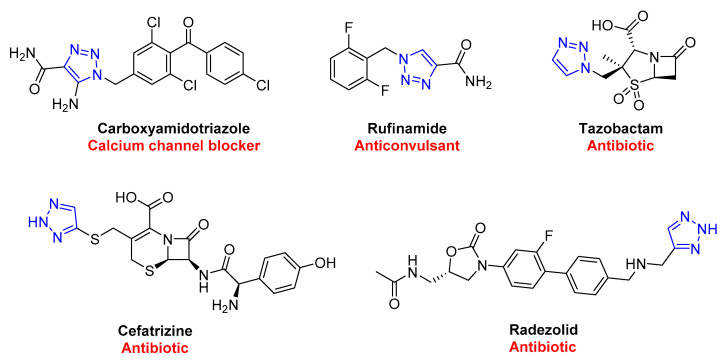
FDA-approved drugs containing 1,2,3-triazole scaffold.

In contrast to the classic thermal (Huisgen) 1,3-dipolar reactions, click chemistry products are generated with high regioselectivity, eliminating the possibility of regioisomers [43,44]. These reactions occur quickly and are thermodynamically driven, as the formation of triazoles is highly exothermic. Click chemistry is also flexible and can tolerate various functional groups, making it easy to synthesize complex molecules. This is helpful in various industries, such as enhancing natural products, polymers, biomolecules, and macrocyclic structures by attaching molecular tags precisely and efficiently [45]. The 1,2,3-triazole scaffold has proven to play a crucial role in the biological properties of various bioactive molecules, showcased in Figure 3, with several FDA-approved drugs containing this scaffold.

## 2. Application of Click Chemistry with Natural Product Hybridization

The integration of click chemistry with natural product modification has opened new avenues for exploring the therapeutic potential of natural products. By attaching molecular tags, functional groups, or probes to natural products through click chemistry, researchers can enhance their properties, improve their stability, facilitate their characterization, and enhance selectivity. The tagged natural products can be utilized in various applications, including drug discovery, target identification, chemical biology, proteomics, and imaging [46,47,48].

### Molecular Hybridization of Alkaloids Using Click Chemistry

Alkaloids are a diverse group of nitrogen-containing secondary metabolites in plants, animals, marine, and microbes [49,50,51,52]. These compounds are typically formed from amino acids and can have a variety of chemical structures. Many alkaloids have been isolated from plants, and have been used to create valuable drugs in modern medicine [53,54,55]. Alkaloids have been shown to have therapeutic properties such as anesthetics, cardioprotective agents, and anti-inflammatory agents. Some well-known alkaloids commonly used in clinical settings include morphine, strychnine, quinine, ephedrine, and nicotine. Recently, there has been a renewed interest in bioactive natural products due to the potential for drug discovery and the development of traditional medicines. Various organic chemistry strategies have extensively explored the click chemistry approach to develop potential drug candidates.

Skiera et al. used cinchona azides and homologated azides to create new 1,2,3-triazole hybrids in combination with antibiotic polyether ionophores, specifically salinomycin and monensin N-propargylamides **1** and **2**. Despite their complex structure, the standard CuAAC protocol was highly efficient in producing the desired products. In vitro testing against three cancer cell lines (LoVo, LoVo/DX, and HepG2) revealed that two compounds had significant antiproliferative activity, with IC_50_ values below 3.00 μM. Furthermore, these compounds were more selective towards normal cells than commonly used anticancer drugs [56].


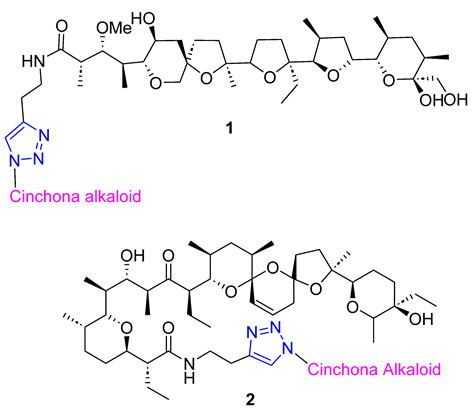


Huang and colleagues successfully created a group of 7-triazole-substituted camptothecin **3** through the CuAAC reaction. The process started with 7-ethynylcamptothecin, which combined 7-chlorocamptothecin with Sonogashira coupling. The alkyne was then combined with different azides using the standard CuAAC protocol, resulting in the corresponding triazoles with high yields. After removing the camptothecin ester, the final products **3** were tested for cytotoxicity against several cell lines, including A549, HCT-116, HT-29, LoVo, and MDA-MB-231. Three products with R = n-Bu, Bn, and −(CH_2_)_4_COOMe showed excellent in vitro activity. Moreover, all of the products **3** maintained their inhibitory ability against Topoisomerase I [57].


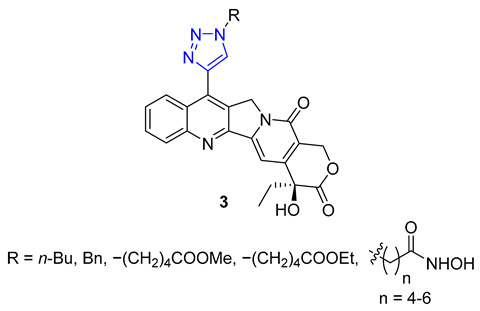


Kuznetsova and Schmalz envisioned making colchicine less toxic and improving its distribution in the body by employing lipophilic groups to the molecule. They used a two-step process that involved reacting colchicine and allocolchicine with propargyl alcohol and then esterifying them with palmitic or oleic acids to create lipophilic triazoles. During testing, they found that only the colchicine derivatives **4** were effective, while the allocolchicine derivatives **5** were less effective. One palmitic ester showed a significant increase in antimitotic activity, but had a lower affinity to the colchicine binding site in tubulin. They then tested these esters by encapsulating them in liposomes made with egg phosphatidylcholine, yeast phosphatidylinositol, and palmitic or oleic esters. The liposomal formulation of the ester with the oleoyl chain was more effective in inhibiting cell proliferation than the unencapsulated ester. Screening these liposomes against four human tumor cell lines confirmed the retained cytotoxicity of colchicine derivatives [58,59].


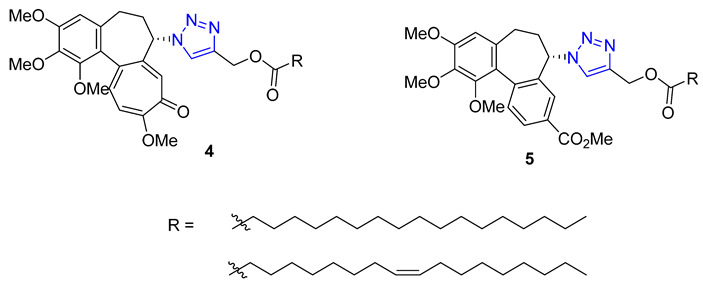


A group of researchers, led by Fedorov, created a set of two-agent combinations called dyads, consisting of colchicine and tubulizine, which are both antimitotic agents. The purpose of this was to enhance their individual anticancer abilities. The process involved using the CuAAC reaction of azide colchicine congeners with acetylene-decorated tubulizine, resulting in products **6** with high yields. All dyads portrayed significant cytotoxic activity against the HBL100 human mammary cell line, with IC_50_ values varying from 0.60 to 2.93 μM. Although all triazoles showed greater activity than tubulizine, none were more effective than deacetylcolchicine. Furthermore, some compounds in this series acted as sub-stoichiometric inhibitors of microtubule assembly [60].


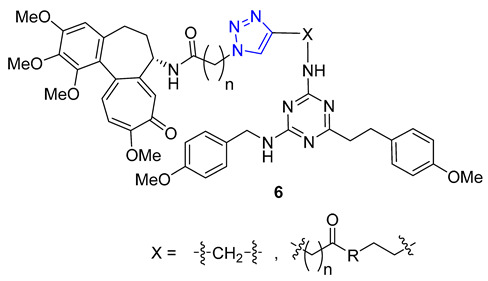


Shi and co-workers utilized berberine as a scaffold to synthesize a novel class of triazole-containing derivatives through click chemistry. The primary objective of this research was to develop potent multivalent inhibitors targeting acetyl- and butyrylcholine esterases (AChE and BChE), as well as to explore their potential as β-amyloid aggregation inhibitors, which play a crucial role in the neurodegenerative cascade of Alzheimer’s disease. The synthetic pathway involved converting berberine azides into partially demethylated berberrubine. Subsequently, *N*-propargyl-substituted tertiary amines were obtained by reacting secondary amines with propargyl bromide. The CuAAC reaction successfully yielded the desired berberine triazoles. Screening of the synthesized compound library led to the identification of several highly active compounds. Compound X (R = 4-diisopropylamine) demonstrated remarkable inhibitory activity against AChE, with an impressive IC_50_ value of 0.044 μM, displaying selectivity towards AChE. Additionally, when the triazole ring bore a dibutylamine substituent, the conjugate exhibited good inhibitory activity against AChE, with an IC_50_ value of 0.20 μM, and displayed the highest potency in inhibiting β-amyloid aggregation [61].


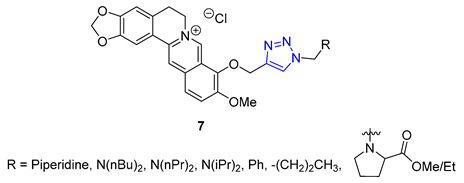


Jin and Yan extended the previous study, focusing on preparing a library of berberine triazoles with reverse connectivity of the triazole group to evaluate their cytotoxicity. O-propargylation of berberrubine was achieved directly, yielding O-propargylberberine, which was subsequently subjected to a CuAAC reaction with substituted benzyl azides to produce the corresponding triazoles. All synthesized products were evaluated against three human cancer cell lines (MCF-7, SW-1990, and SMMC-7721) and the HUVEC line (normal human umbilical vein endothelial cell) to assess their potential as anticancer agents. Remarkably, most of the derivatives displayed significantly higher anticancer activities against MCF-7 cells than the parent berberine. Compound **8**, featuring a 4-tert-butyl substituent in the phenyl ring, exhibited the highest potency against the SW-1990 and SMMC-7721 cell lines, with IC_50_ values of 8.5 and 11.9 μM, respectively [62].


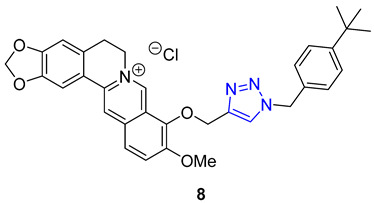


Pingaew et al. synthesized a novel 1,2,3,4-tetrahydroisoquinoline derivative containing a 1,2,3-triazole moiety using a modified Pictet–Spengler reaction and the CuAAC method to introduce the triazole ring. The antiproliferative activity of the products was evaluated against four cancer cell lines (HuCCA-1, HepG2, A549, and MOLT-3), and some compounds (**9**) displayed significant cytotoxicity. Notably, compounds with R = *o*-aryl methyl ester demonstrated exceptional potency against HuCCA-1 (IC_50_ = 0.63 μM) and A549 (IC_50_ = 0.57 μM) cell lines. Additionally, triazole X (R = *p*-Toluenyl) exhibited remarkable activity against HepG2 cells (IC_50_ = 0.56 μM), and surpassed the activity of drugs such as etoposide or doxorubicin without harming normal cells [63].


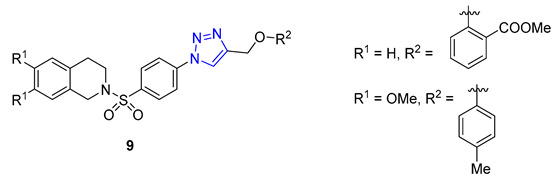


Researchers led by Melander tackled the challenge of fighting biofilm-forming bacteria, which are responsible for 75% of human body infections. They turned to the flustramine family of alkaloids to combat this issue, known for their broad antimicrobial activity. The researchers used them as a scaffold for synthesizing an 18-member library of pyrroloindoline 1,2,3-triazole amides through CuAAC synthesis. The team created the library by reacting various azides with a synthetic alkyne-functionalized pyrroloindoline mimic of flustramine, then deprotecting the pyrrolidine nitrogen atom. The library was then screened against Gram-positive and Gram-negative bacterial strains, including *A. baumannii*, *E. coli*, and MRSA (methicillin-resistant *Staphylococcus aureus*), to determine its ability to modulate biofilm formation. The screening resulted in the identification of several nontoxic compounds with low micromolar IC_50_ values. Compound **10** showed high activity against MRSA with p-alkylphenyl groups (C5–C7). It effectively inhibited biofilm formation in methicillin-sensitive strain *Staphylococcus aureus* with IC_50_ values ranging from 6.6 to 32.0 μM [64].


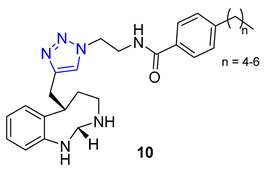


Ochrolifuanine E, a *bis*-indole alkaloid found in the Thai herbal medicine plant *Dyera costulata*, emerged as a potential inhibitor of β-secretase (BACE1) through a virtual screening procedure conducted by Vajragupta’s group. BACE1 plays a critical role in producing amyloid peptides from the amyloid precursor protein, making it a promising target for Alzheimer’s disease drug development. Docking studies of the parent alkaloid revealed that tryptoline (2,3,4,9-tetrahydro-1H-pyrido [3,4-b]indole) served as the key pharmacophore responsible for the enzyme binding. This insight led to the design of a library of 1,2,3-triazolyl tryptoline derivatives from which 22 most promising candidates, selected based on docking analysis, were synthesized using the CuAAC reaction. Screening of these derivatives for inhibitory activity against BACE1 resulted in the identification of a potent inhibitor, compound **11** (IC_50_ = 1.49 μM), exhibiting 100 times greater selectivity to BACE1 over Catepsin D [65].


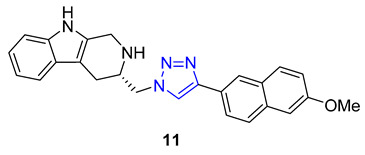


Shinde and colleagues presented a new approach to creating 1,2,3-triazole-substituted piperidines. They achieved this by adding an azido group to the fourth position of the piperidine ring, using a simple transformation of N-Boc-protected piperidinone. The resulting azide was then combined with either ethyl propiolate or propargyl alcohol, using CuI as a catalyst to produce the desired products. These products were then further modified to create derivatives **12** and **13**, exhibiting exceptional antifungal properties [66,67].


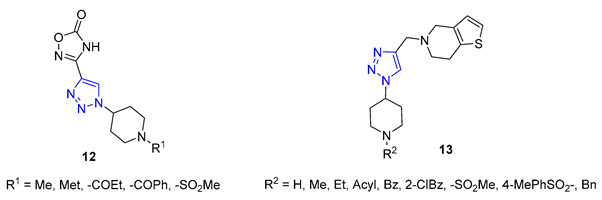


Da Silva utilized 6-mercaptopurine, a synthetic drug that is similar to naturally occurring purine alkaloids, to create a new series of mono- and bis-1,2,3-triazolyl derivatives. He first conducted N- and S-propargylation of 6-mercaptopurine to produce mono- or dipropargyl derivatives. These were then subjected to the CuAAC reaction with azidoacetic acid and methyl 3-β-azidocholanoate. From a range of products, compounds **14** and **15** demonstrated exceptional antimalarial activity in vitro, with a higher rate of parasite multiplication inhibition compared to the common antimalarial drug, chloroquine [68].


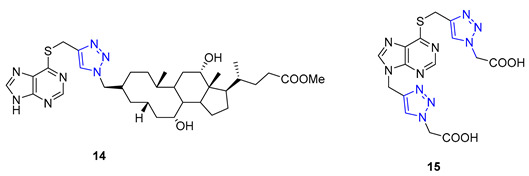


In a recent study, Nair and colleagues explored the potential of purine as a building block for developing novel neuroprotective agents. Their approach involved integrating a 1,2,3-triazole ring and fluoroaromatic groups into the purine scaffold. To begin, the researchers modified commercially available 2,6-dichloropurine by introducing an alkyne group through a two-step process. They then utilized the CuAAC reaction within *situ*-generated benzyl azides to construct a series of compounds, including Compound **16**. These compounds were evaluated for their ability to safeguard neurons using fluorescence electron microscopy. Interestingly, one of the derivatives containing an *o*-fluorophenylmethyl group exhibited neuroprotective properties on par with flavopiridol and roscovitine, two well-known cyclin-dependent kinase (CDK) inhibitors. This compound effectively mitigated amyloid β (Aβ)-induced neurotoxicity [69].


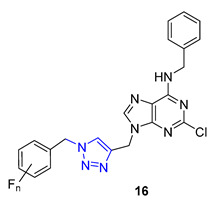


The Wang group developed glycoconjugates of phenanthroindolizidine alkaloids that target tobacco mosaic virus (TMV). The team utilized three different methods to conjugate (S)-6-O-desmethylantofine and 14-hydroxytylophorine with sugars. One of the methods involved creating a 1,2,3-triazole linker to attach O-propargylated alkaloids with three different monosugar units. The resulting glycoconjugates, **17** and **18**, demonstrated improved water solubility. However, their activity was only moderate compared to glycoconjugates linked by simple glycosidic bonds, which showed highly active results. These findings highlight the significance of the glycosidic bond in creating potent activity in glycoconjugates that target TMV [70].


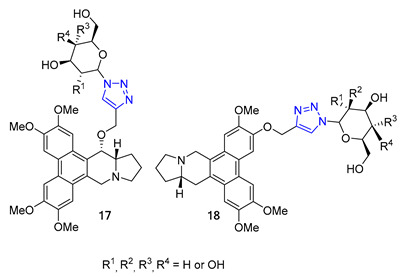


Carroll and their team have effectively created a range of cocaine-like compounds called 2β-alkynyl and 2β-(1,2,3-triazolyl)-3β-(aryl)tropanes. They tested the compounds’ ability to bind to dopamine, serotonin, and norepinephrine membrane transporters using radioligand binding assays. Surprisingly, all of the substances produced from the alkyne intermediate through the CuAAC reaction, including two triazoles designated as **19,** demonstrated a strong affinity for the dopamine transporter (DAT) in nanomolar or sub-nanomolar concentrations [71].


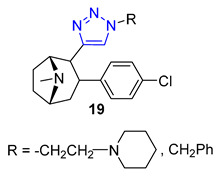


Researchers Thuy et al. have investigated the properties of Murrayafoline A, a carbazole alkaloid found in the roots of different Murraya species. This substance has a wide range of biological activities, including the ability to kill fungi and induce apoptosis. To explore its potential further, the researchers synthesized a series of derivatives called X by combining the azido derivative of Murrayafoline A with various alkynes using a CuAAC reaction. They then evaluated the biological effects of these derivatives, focusing on their anti-inflammatory properties. The results showed that two of the triazoles **20** (R = −CH_2_NH_2_ and −CH_2_OH) were particularly effective at inhibiting the production of cytokines IL-12 p40, IL-6, and TNF-α, outperforming the unmodified Murrayafoline A [72].


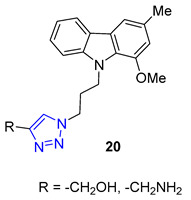


Matrine, a quinolizidine alkaloid derived from the root of *Sophora flavescens Ait* (also known as Kushen), is a traditional Chinese herb used for centuries for the treatment of liver disease. Zhao et al. focused on synthesizing matrine–triazole–chalcone hybrids and reported their notable growth inhibitory effects on a range of cancer cells. Compound **21** (IC_50_ = 5.01–7.31 μM) exhibited broad-spectrum anticancer activities against various cancer cell lines (A549, Bel-7402, Hela, and MCF-7). Notably, Compound **21** displayed superior potency compared to the combination of matrine and chalcone (IC_50_ > 50 μM) as well as 5-fluorouracil (IC_50_ = 8.93–40.38 μM). Structure–activity relationship (SAR) studies suggested that the α, β-unsaturated ketone moiety and the triazole played crucial roles in determining the enhanced inhibitory activity. Further investigations revealed compound **21** could induce apoptosis in A549 cells and effectively suppress tumor growth in an A549-xenografted nude mouse model (at 10 mg/kg) without causing apparent cytotoxicity [73].


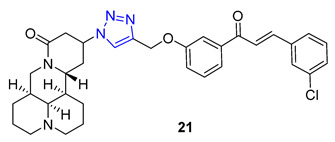


Homoharringtonine and homoerythrina, naturally occurring alkaloids isolated from the genus Cephalotaxus, have gained significant attention due to their derivatives’ diverse biological activities, particularly their potential as anticancer agents. In a study conducted by Li et al., homoerythrina–triazole **22** (IC_50_ = 1.89–4.19 μM) displayed more potent inhibitory activity than rucaparib (IC_50_ = 4.91–13.51 μM) and harringtonine (IC_50_: 10.55–11.71 μM) in A549, HCT-116, and MCF-7 cancer cells. Mechanistic investigations revealed that **22** arrested the cell cycle at the S phase, inhibited PAR biosynthesis, and induced apoptosis in A549 cells. Additionally, the same research group reported erythrina–triazole **23** (IC_50_ = 0.23–1.13 μM) to exhibit superior inhibitory activity compared to rucaparib (IC_50_ = 2.58–13.82 μM) across a panel of cancer cell lines (A549, OVCAR-3, HepG2, A375, and SW-620). Mechanistic studies indicated Compound **23** inhibited PAR biosynthesis and induced apoptosis in A549 cells [74,75].


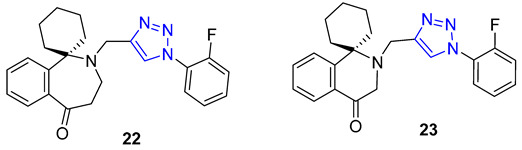


Combining two different natural products via the CuAAC reaction has proven to be an effective method for creating new, functional compounds. One such compound is cytisine–triazole–camphor **24**, reported by Artyushin et al., which was found to have strong antiviral properties against influenza virus A/Puerto Rico/8/34 (H1N1), with low toxicity and an impressive selectivity index (IC_50_ = 8 μM, CC_50_ = 168 μM, SI = 20). Notably, its selectivity index is higher than that of the reference drug rimantadine (IC_50_ = 67 μM, CC_50_ = 335 μM, SI = 5) [76].


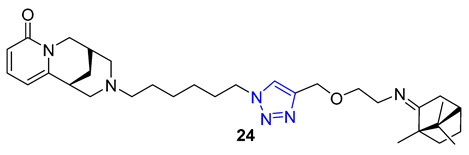


Theophylline, a naturally occurring purine base, serves as a bronchodilator drug for the treatment of respiratory diseases such as chronic pulmonary obstructive disease and asthma. Ruddarraju et al. sought to explore its antibacterial properties in vitro by linking theophylline and nucleosides by incorporating a triazole ring using conventional Huisgen conditions. The triazole-tethered theophylline–nucleoside hybrid **25** exhibited significant growth inhibition against A549, HT-29, MCF-7, and A375 cancer cells (IC_50_ = 1.89–4.89 μM). On the other hand, hybrid **26** displayed potent antibacterial activities against both Gram-positive strains (*Staphylococcus aureus*, *Bacillus cereus*) and Gram-negative strains (*Escherichia coli* and *Pseudomonas aeruginosa*) with impressive MIC values (MIC = 0.03125–0.125 μg/mL), comparable to or even more potent than the clinical drug ciprofloxacin (MIC = 0.0156–0.0625 μg/mL) [77,78].


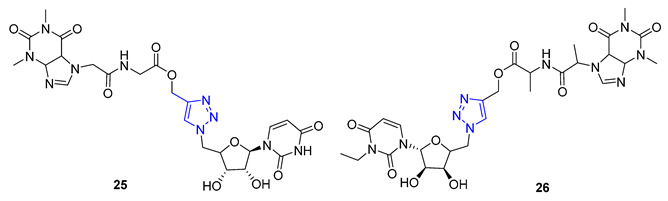


Quinine, a natural cinchona alkaloid, is a crucial element in antimalarial drugs and is widely accessible. Boratyński et al. recently created a range of quinine derivatives by introducing azide at C9, C2’, and C6’. This resulted in a focused library of triazole-containing chinchona alkaloids (**27**–**30**). In vitro studies revealed that most of these derivatives possessed moderate antiproliferative activity. Among them, **27** (IC_50_ = 0.53 μM) displayed the most potential in MC-4-11 cells, while **28** (IC_50_ = 1.2 μM) was most effective in HT-29 cells [79].


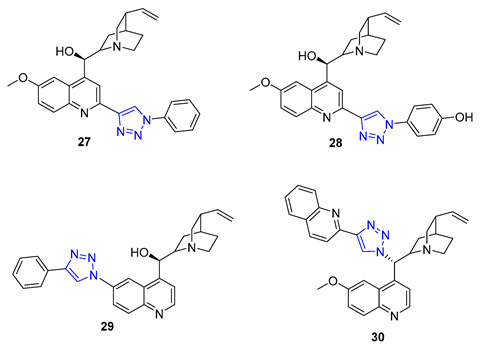


New molecules can have unexpected properties when small molecules are combined with ferrocene, a unit known for its adjustable redox characteristics. Pešić et al. discovered that **31** with IC_50_ value 2.34–2.13 μM, a ferrocene–quinine conjugate, inhibited the growth of both drug-sensitive NCI-H460 cancer cells and multi-drug resistant (MDR) NCI-H460/R cancer cells. Mechanistic studies showed that **31** increased ROS production and induced mitochondrial damage in MDR cancer cells, highlighting the importance of the ferrocene compound [80]. Meanwhile, Sahu et al. reported that **32**, a C19 quinine–triazole derivative, had potent antimalarial with IC_50_ = 0.25 μM against P. falciparum and antileishmanial activities (*L. donavani*, IC_50_ = 1.78 μM) with no apparent adverse effects. Structural toxicological activity relationship studies suggested that including the triazole compound in quinine decreased toxicity [80]. Additionally, Panda et al. found that **33** exhibited more potent in vitro antimalarial activity with an IC_50_ value of 27 nM than quinine (IC_50_ = 58 nM) against P. falciparum strain 3D7, likely due to the addition of a hydrophobic alkyl chain at C9 that improved the scaffold’s penetration ability [81].


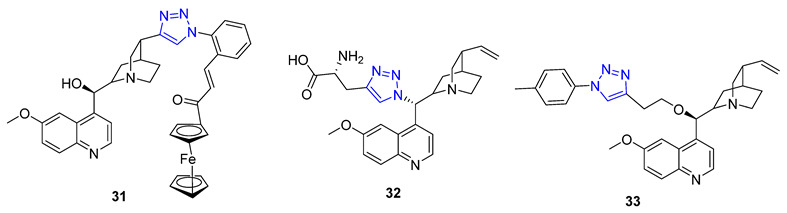


Berberine is a type of isoquinoline alkaloid that can be found in different Berberis plants. Berberine–triazoles have been found to possess both anticancer and antimalarial properties. In a study conducted by Sun et al., it was discovered that a compound called **34** could inhibit the growth of SW-1990 (IC_50_ = 22.2 μM) and SMMC-7721 (IC_50_ = 14.9 μM) cancer cells [82]. Meanwhile, Nath et al. reported that berberine–triazole **35** (IC50 = 0.142 μM) could exert antimalarial activity against the P. falciparum (3D7) strain without causing any apparent harm to human PC-3 cells (IC_50_ > 200 μg/mL) [83].


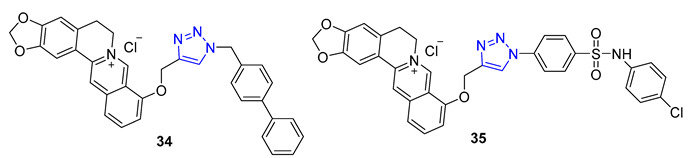


In 1966, Camptotheca acuminata yielded 20(S)-Camptothecin, a powerful inhibitor of DNA topoisomerase I. Xu and colleagues later synthesized a range of C10 homocamptothecin–triazole derivatives by introducing an alkyne at C10 of homocamptothecin and conducting reactions with various azides under CuAAC. Among them, derivative **36** (IC_50_ = 30 nM) was found to be more effective at inhibiting A549 cancer cells in a Topo I-dependent manner than 20(S)-camptothecin (IC_50_ = 170 nM). According to mechanistic studies, **35** can halt the cell cycle at G2 and S phases [84].


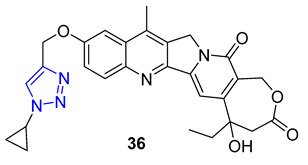


## 3. Drug-like Properties

Alkaloids, like vinblastine, were originally discovered in plants and have been approved for cancer treatment. However, their effectiveness is limited by poor solubility, low bioavailability, drug resistance, and possible liver toxicity [85,86,87]. This has hindered further experimentation beyond lab testing. Therefore, it is crucial to thoroughly understand the drug-like properties of alkaloids when developing potential drug candidates and using them as scaffolds. Preclinical drug development involves ADMET studies, which stands for Absorption, Distribution, Metabolism, Excretion, and Toxicity studies. These studies evaluate a drug candidate’s pharmacokinetic and pharmacodynamic properties. ADMET studies provide essential information about a drug’s efficacy, safety, and pharmacokinetic properties, which helps make informed decisions regarding dosage, formulation, and potential risks associated with the drug candidate. Integrating ADMET studies into natural product drug development provides researchers with valuable insights into these compounds’ pharmacokinetic and pharmacodynamic properties, which helps demonstrate their drug-like properties [88,89,90,91,92,93,94].

A molecule’s drugability can be estimated using Lipinski’s rule of five (RO5), determining if a biologically active chemical is bioavailable [95,96]. The rule states that certain molecular qualities, such as having no more than five hydrogen bond donors, a maximum of ten hydrogen bond acceptors, a mass of less than 500 Da, and a partition coefficient (logP) value of less than five, are linked to pharmacokinetic drug features such as absorption, distribution, metabolism, and excretion. If one or more of these conditions are violated, the molecule is predicted to be incapable of being taken orally. The number of rotatable bonds and polar surface areas are also significant factors in assessing oral bioavailability [97]. For example, the maximum number of rotatable bonds should be ten, and a molecule’s polar surface area should be no larger than 140 Å for orally active medicines carried by the transcellular route [98,99]. The hERG IC_50_ value is another critical characteristic determining a drug’s effectiveness in blocking potassium hERG channels. An ideal value for hERG pIC_50_ is less than five for consideration as a safer drug candidate [100,101,102,103]. The computational tools STARDROP and SwissADME were used to determine the ADMET and drug-like properties [104,105], compiled in Table 1, and can aid the development of effective and safer drug candidates.

In this article, we have compiled data on the common drug-like properties of potential molecularly hybridized alkaloids using click chemistry. Most of these compounds meet the required parameters; however, some (highlighted in red) have potential biological properties but do not meet the recommended values of fundamental drug-like properties.

## 4. Clinical Trials

The pharmacological benefits of alkaloids have garnered increasing attention of late. Extensive clinical studies have explored their efficacy in addressing various conditions, including inflammation, skin conditions, constipation, Alzheimer’s disease, gastrointestinal issues, respiratory problems, liver problems, diabetes, cancer, neurotoxicity, schizophrenia, and kidney disease [106]. Over 135 clinical studies have been conducted on alkaloids, and several more compounds based on alkaloids have entered clinical trials.

## 5. Conclusions and Future Perspectives

Accessing a varied assortment of efficient molecules is paramount in developing new pharmaceuticals with specific biological functions. Click chemistry has proven useful in facilitating drug discovery and enabling access to many bio-diverse molecules. Alkaloids, owing to their diverse biological activities, intricate structures, and natural abundance, present themselves as promising prospects for contemporary drug discovery. Modifying alkaloids is necessary to optimize their drawbacks and create natural product-like drug screening libraries. Therefore, synthetic toolboxes enabling efficient access to molecular hybridization and unique natural product functions are highly desirable. Click chemistry is one of the most effective approaches for synthesizing diverse natural product derivatives. Adding 1,2,3-triazole via click chemistry and molecular hybridization can enhance biological activity and improve kinetics and drug-like properties. Many of these derivatives have shown new functions and could serve as an inexhaustible source for discoveries in drug development. However, several issues and new research directions still need to be addressed to fully harness the power of click chemistry in natural product-based drug discovery. According to computational ADMET studies, most hybridized alkaloids are toxic, with hERG pIC_50_ values exceeding the acceptable limit. Our review article will provide valuable guidance to researchers on alkaloid-based drug development.

## Figures and Tables

**Table 1:** ADMET and drug-like properties of potential molecularly hybridized alkaloids (**bold red** numbers indicates violation of recommended key drug-like properties).

Compd.	logP	MW	HBD	HBA	TPSA	Rotatable Bonds	hERG pIC_50_	BBB	HIA
**8**	**6.11**	**549.6**	0	8	71.5	7	**6.47**	-	+
**11**	4.21	409.5	2	6	67.8	4	**6.26**	-	+
**14**	**5.25**	653.9	3	**11**	**151.9**	10	**5.82**	-	-
**15**	0.36	430.4	2	**14**	179.6	9	3.62	-	-
**20a**	3.00	350.4	1	6	65.1	6	**5.99**	-	+
**20b**	2.72	349.4	1	6	70.9	6	**5.80**	-	+
**21**	**5.01**	**586.1**	0	8	80.6	7	**6.74**	-	+
**22**	3.69	404.5	0	5	51.0	3	**6.77**	-	+
**23**	3.54	390.5	0	5	51.0	3	**6.64**	-	+
**24**	**5.05**	**548.8**	0	8	77.5	**12**	**6.58**	-	+
**25**	−0.77	**660.6**	3	**20**	236	**12**	3.11	-	+
**26**	−2.17	**604.5**	4	**20**	**246.9**	**11**	2.76	-	+
**27**	3.70	467.6	1	7	76.3	6	**6.59**	-	+
**28**	3.28	483.6	2	8	96.5	6	**6.59**	-	+
**29**	4.27	436.5	1	5	54.2	5	**6.74**	-	+
**30**	4.50	**502.6**	0	7	68.9	6	**7.47**	-	+
**32**	0.20	462.5	2	9	119.4	8	**5.07**	-	-
**33**	**5.18**	**509.6**	0	7	65.3	9	**7.41**	-	+
**34**	**6.26**	**539.6**	0	7	62.28	6	**6.60**	-	-
**35**	**5.51**	**639.1**	1	10	108.4	7	**7.09**	-	+
**36**	2.51	**513.5**	1	10	121.4	5	**5.01**	-	+
